# Pharmacological screening and transcriptomic functional analyses identify a synergistic interaction between dasatinib and olaparib in triple‐negative breast cancer

**DOI:** 10.1111/jcmm.14980

**Published:** 2020-02-07

**Authors:** Verónica Corrales‐Sánchez, María del Mar Noblejas‐López, Cristina Nieto‐Jiménez, Javier Pérez‐Peña, Juan Carlos Montero, Miguel Burgos, Eva M. Galán‐Moya, Atanasio Pandiella, Alberto Ocaña

**Affiliations:** ^1^ Translational Research Unit Albacete University Hospital Albacete Spain; ^2^ Translational Oncology Laboratory Centro Regional de Investigaciones Biomédicas (CRIB) Universidad de Castilla La Mancha Albacete Spain; ^3^ Instituto de Biología Molecular y Celular del Cáncer (IBMCC‐CIC) Salamanca Spain; ^4^ IBSAL Salamanca Spain; ^5^ CIBERONC Salamanca Spain; ^6^ CSIC Salamanca Spain; ^7^ Unidad de nuevas terapias y Oncología traslacional IDISSC and CIBERONC Hospital Clínico Universitario San Carlos Madrid Spain

**Keywords:** breast cancer, dasatinib, olaparib, screening, triple negative

## Abstract

Identification of druggable vulnerabilities is a main objective in triple‐negative breast cancer (TNBC), where no curative therapies exist. Gene set enrichment analyses (GSEA) and a pharmacological evaluation using a library of compounds were used to select potential druggable combinations. MTT and studies with semi‐solid media were performed to explore the activity of the combinations. TNBC cell lines (MDAMB‐231, BT549, HS‐578T and HCC3153) and an additional panel of 16 cell lines were used to assess the activity of the two compounds. Flow cytometry experiments and biochemical studies were also performed to explore the mechanism of action. GSEA were performed using several data sets (GSE21422, GSE26910, GSE3744, GSE65194 and GSE42568), and more than 35 compounds against the identified functions were evaluated to discover druggable opportunities. Analyses done with the Chou and Talalay algorithm confirmed the synergy of dasatinib and olaparib. The combination of both agents significantly induced apoptosis in a caspase‐dependent manner and revealed a pleotropic effect on cell cycle: Dasatinib arrested cells in G0/G1 and olaparib in G2/M. Dasatinib inhibited pChk1 and induced DNA damage measured by pH2AX, and olaparib increased pH3. Finally, the effect of the combination was also evaluated in a panel of 18 cell lines representative of the most frequent solid tumours, observing a particularly synergism in ovarian cancer. Breast cancer, triple negative, dasatinib, olaparib, screening.

## 1 INTRODUCTION

Targeting DNA repair mechanisms in cancer cells is a novel therapeutic approach that has reached the clinical setting in several tumours, including ovarian and triple‐negative breast cancer.^1^, ^2^, ^3^

Some cancer cells lack mechanisms involved in DNA repair, such as when inactive mutations of the BRCA1 and BRCA2 genes impair the adequate restoration of DNA lesions through the homologous recombination (HR) pathway, what subsequently produces a high grade of genomic instability.^1^, ^2^ In tumours with deficits in the HR mechanism, other complementary pathways are used by cells. For instance, the non‐homologous end joining (NHEJ), where the protein PARP1 plays a central role.^4^ Inhibition of PARP enhances the activity of DNA‐damaging agents in tumours lacking functional BRCA1 and BRCA2 genes, through a synthetic lethality interaction.^2^, ^4^, ^5^ In this context, PARP inhibitors have reached the clinical setting, as is the case for olaparib, approved in ovarian and breast cancers with germline mutations of the BRCA gene, and therefore impairment of the HR repair pathway.^6^, ^7^, ^8^ Of note, other PARP inhibitors like rucaparib and niraparib have recently shown activity in ovarian cancer gaining regulatory approval.^9^, ^10^

PARP inhibitors have shown to be active when given alone, but also, as mentioned, when combined with agents that produce DNA damage, like platinum derivatives.^11^, ^12^ However, the main limitation of these combinations is the high associated toxicity, mainly bone marrow suppression, what clearly limits their clinical development.^11^, ^12^, ^13^ In this context, the identification of other synthetic lethality interactions that could augment the efficacy of PARP inhibitors without adding toxicity is a main objective.

Triple‐negative breast cancer is characterized by a high grade of genomic instability, and a greater presence of mutations at genes involved in DNA repair pathways. In addition, it is associated with detrimental clinical outcome, and a limited number of therapeutic options for patients exist.^14^, ^15^

By using a system biology approach followed by a pharmacological screening, we identified relevant druggable functions in TNBC. We observed that the multikinase inhibitor dasatinib was synergistic with PARP inhibition in triple‐negative cell lines. Dasatinib alone inhibited Chk1, induced DNA damage and arrested cells in G1, meanwhile olaparib arrested cells in G2/M. The combination significantly induced cell death in a caspase‐dependent manner and showed relevant activity in other tumour types, like ovarian cancer. Taken together, our results pave the way for the clinical development of this combination in triple‐negative breast and ovarian cancer.

## 2 MATERIAL AND METHODS

### 2.1 Cell culture and drug compounds

Triple‐negative breast cancer cell lines MDA‐MB‐231, HS‐578T and BT549 were growth in DMEM and HCC3153 was growth in RPMI. Ovarian cancer, SKOV3; prostate cancer, DU145; colorectal cancer, HT29; head and neck squamous carcinoma, SCC2, SCC40 and SCC38; lung cancer, A549, H727 and H1299 and no TNBC breast cancer, BT474 and MCF7; cell lines were growth in DMEM. Ovarian cancer, OVCAR3, OVCAR8 and IGROV1; prostate cancer, PC3, and colorectal cancer, SW620 and SW480, cell lines were growth in RPMI. Normal epithelial cell line, HACAT, was growth in DMEM. The BRCA1/2 mutation status was obtained from the cancer cell line Encyclopedia (https://portals.broadinstitute.org/ccle).

All medium contained a high glucose concentration (4.5 g/L) and were supplemented with inactivated foetal bovine serum (10% FBS), antibiotics (100 U/mL penicillin and 100 μg/mL streptomycin) and L‐glutamine (2 mmol/L). All cells were growth on adherent culture and maintained at 37ºC in a humidified atmosphere in the presence of 5% CO_2_. All cell lines used were provided by Drs. J. Losada and A. Balmain (from the ATCC) in 2015. Cells were authenticity was corroborated annually by STR analysis at the molecular biology unit at the Salamanca University Hospital.

All cell culture mediums and supplements were obtained from Gibco (Thermo Fisher) and Sigma‐Aldrich. Src inhibitor, dasatinib and PARP inhibitor, olaparib were purchased by Selleckchem.

### 2.2 Whole‐genome transcription profiling and gene set enrichment analyses

mRNA level data from normal breast tissues and tumour (basal‐like, Luminal A, Luminal B and HER2+) tissues were extracted from public data sets (GEO DataSet accession numbers: GSE21422, GSE26910, GSE3744, GSE65194 and GSE42568). From these studies, only those samples that meet clearly the requirements to be included in some of the breast cancer subtypes were included in the analysis (Table S1). Affymetrix CEL files were downloaded, normalized and analysed using the RMA algorithm implemented in the Affymetrix Expression Console. We further performed gene set enrichment analysis (GSEA) to identify differential gene sets, associated with a range of cell functions, between normal and tumour tissues. 178 gene sets were collected from the Molecular Signatures Database (MSigDB) (http://www.broadinstitute.org/gsea/msigdb/), including 16 from REACTOME, four from Kyoto Encyclopedia of Genes and Genomes (KEGG), three from BioCarta Pathways, 109 from Gene Ontology (GO) biological process and six from Pathway Interaction Database (PID) via the NDEx database (http://www.ndexbio.org), among others; data were analysed by GSEA with parameter set to 1000 gene set permutations. Enrichment score corresponds to a weighted Kolmogorov‐Smirnov‐like statistic and reflects the extent to which the gene set is overrepresented at the extreme (ie top or bottom) of the entire ranked list. If the enrichment score is positive (eg the gene set is overrepresented by top ranked genes), then the gene set is considered up‐regulated, while it is considered down‐regulated if the score is negative. The network of gene sets interactions was constructed by using Cytoscape software (version 3.4.0).

### 2.3 Cell proliferation studies: MTT assays for single drug and drug combination treatments

MTT assay is a colorimetric assay that allow to validate the capacity of the cytotoxic agents used through the assessment of the cell mitochondrial activity. The compound 3‐ (4.5‐Dimetill‐2‐thiazolyl) −2.5‐diphenyl‐2H‐tetrazolium bromide (MTT) is metabolized by the enzyme mitochondrial succinate dehydrogenase to coloured formazan salts. The amount of formazan produced is associated with the number of metabolically active cells. Antiproliferation effect quantification will be proportional to the absorbance of the medium.

Cells (10 000 cells/well, 48‐multiwell plates) were treated with each drug (100 nmol/L) for 72 hours. Then, drug‐containing medium was replaced with red phenol‐free DMEM containing MTT (0.5 μg/μL) and cells were incubated in the dark for 45 minutes at 37°C. To solubilize the formazan salts generated, MTT solution was removed and dimethyl sulfoxide (DMSO) (Fisher‐Scientifics) was added to the wells. Absorbance values (A_555_ nm ‐ A_690_ nm) were recorded in a multiwell plate reader (BMG labtech). Same approached was followed for drug combination analyses.

To determine EC_50_ values, we used increasing concentrations of dasatinib and olaparib (72 hours). EC_50_ values were calculated with GraphPad software. For synergy studies, we used combination doses dasatinib and olaparib in a constant ratio. To determinate which combinations were synergistic, additive or antagonic, we used the Calcusyn 2.0 software (Biosoft). The mathematical expression used is the Chou‐Talalay algorithm, which allows to obtain the combination index (CI) indicating synergistic effect (<1), additive effect (=1) and antagonistic effect (>1).

### 2.4 Aggressiveness studies: Matrigel, clonogenic and soft agar assays

MDA‐MB‐231 and HS‐578T (5,000 cells) were resuspended in culture medium with Matrigel (Sigma‐Aldrich, 2%) and seeded on a thick layer (1 mm) of matrigel (48‐wells plates). After 24 hours of incubation, MDA‐MB‐231 and HS‐578T invading cultures were treated with dasatinib (100 nmol/L) and olaparib (1 nmol/L and 2 nmol/L and 10 nmol/L and 20 nmol/L, respectively) alone and in combination. Invading cultures growth was monitored daily in an inverted microscope to evaluate cell morphology.

MDA‐MB‐231 and HS‐578T cells (100.000 cells/p6 well) were treated with dasatinib (100 and 250 nmol/L, respectively), olaparib (5 and 50 μmol/L, respectively), alone or in combination. After 24 hours of treatment, MDA‐MB‐231 (500 cells) and HS‐578T (1000 cells) were reseeded in p6 wells in triplicates by each condition. Seven days later, cells were fixed with glutaraldehyde (0.1% in H_2_O, 15 minutes) and stained with crystal violet (0.05% in H_2_O, 15 minutes). The number of colonies was counted by Image J software. The results were referred to the control as percentage.

For soft agar assays, cells (10 000/6‐well culture plates) were seeded on an agarose layer (0,4%), which was on top of a base agar layer (0,8%). Then, cells were treated at indicated doses of drugs. Cells were incubated for 21 days at 37°C and 5% CO_2_. Cell colony formation was then examined under a light microscope after staining with 0.05% Crystal violet.

### 2.5 Cell death and cell cycle analysis: Flow cytometry studies

MDA‐MB‐231 and HS‐578T cells (200.000 cells/plate in 60 mm dishes) were treated with olaparib (2.5 and 5 μmol/L, and 25 and 50 μmol/L, respectively), dasatinib (100 and 250 nmol/L, and 50 and 100 nmol/L, respectively) alone or in combination. Final analysis was performed on a FACSCanto™ II flow cytometer (BDBiosciences).

For cell cycle analysis, after 24 hours of treatment, cells were collected, washed with PBS, fixed in 70% ethanol for 30 minutes in ice cold and then, centrifuged at 4000 rpm/5 minutes. Pellets were washed in PBS containing BSA at 2% and incubated with Propidium iodide/RNAse staining solution (Immunostep SL) for 1 hour at 4°C in the dark.

For cell death studies, after 72 hours of treatment, adherent and floating cells were collected, washed with PBS and then, stained with 2.5 μL of Annexin V‐DT‐634 (AV) (Immunostep SL) and 3 μL of Propidium iodide (PI) (10 mg/mL) in 1x Annexin Binding Buffer (10 mmol/L HEPES, pH 7.4, 140 mmol/L NaOH, 2.5 mmol/L CaCl2) for 1 hour at room temperature in dark conditions. Analysis distinguished living cells (AV‐, PI‐) vs apoptotic cells (AV+, PI‐ and AV+, PI+).

For caspase assay, cells were seeded at the conditions described above. MDA‐MB‐231 and HS‐578T cells were treated with olaparib (5 μmol/L and 50 μmol/L, respectively), dasatinib (50 nmol/L and 250 nmol/L, respectively) alone or in combination. Prior to drug exposure, cells were culture in presence or absence of pan‐caspase inhibitor QVD (10 μmol/L). QVD was maintained during all drug treatment.

### 2.6 Preparation of cell extracts and western blotting

After treatment, cells were washed with phosphate‐buffered saline (PBS) (137 mmol/L NaCl, 2.7 mmol/L KCl, 8 mmol/L Na_2_HPO_4_, 1.5 mmol/L KH_2_PO_4_) and lysed in ice‐cold lysis buffer (20 mmol/L Tris–HCl [pH 7.0], 140 mmol/L NaCl, 50 mmol/L EDTA, 10% glycerol, 1% Nonidet P‐40, 1 μmol/L pepstatin, 1 μg/mL aprotinin, 1 μg/mL leupeptin, 1 mmol/L phenylmethyl sulfonyl fluoride, 1 mmol/L sodium orthovanadate). Then, insoluble material was removed by centrifugation. The protein concentration was determined using BCA (Bicinchoninic acid) protein assay kit (Sigma‐Aldrich). About 50‐80 μg protein were boiled in electrophoresis sample buffer and placed on 6%–15% sodium dodecyl sulfate–polyacrylamide gel electrophoresis (SDS‐PAGE), depending on the molecular weight of the proteins to be analysed. After electrophoresis, proteins in gels were transferred to polyvinylidene difluoride membranes (Millipore Corporation). Membranes were blocked in Tris‐buffered saline with Tween (TBST) (100 mmol/L Tris [pH 7.5], 150 mmol/L NaCl, 0.05% Tween 20) containing 1% of bovine serum albumin or 5% of skimmed milk for 1 hour and then incubated for 2‐16 hours with the following primary human monoclonal/polyclonal antibodies: anti‐GAPDH, anti‐cyclin B, (purchased from Santa Cruz Biotechnology), anti‐PARP, anti‐p(Y15)CDK1, anti‐p27, anti‐cyclin D1, anti‐Chk1, anti‐pChk1, anti‐pChk2, anti‐pH2AX, anti‐Src, anti‐p‐Src (obtained from Cell Signalling Technologies), anti‐pHistone H3 (purchased from Millipore Corporation) and anti‐Calnexin (obtained from Stressgen Bioreagents). After washing with TBST, membranes were incubated with HRP‐conjugated antimouse or anti‐rabbit secondary antibodies for 30 minutes and bands were visualized by using ECL Plus Western blotting Detection System (GE Healthcare).

### 2.7 Statistical analysis

All experiments were designed using triplicates for each condition and were performed at least three times. We used *t* test for independent samples non‐parametric assay, together with the Levenne test to consider, or not, equal variances. The level of significance was considered 95%; therefore, *P* values lower than .05 were considered statistically significant: *P ≤ .05; **P ≤ .01 and ***P ≤ .001. Software SPSS version 22 (IBM SPSS Statistics) was used.

## 3 RESULTS

### 3.1 In silico analyses and pharmacological screening identify druggable functions and active compounds in TNBC

**Figure 1:**
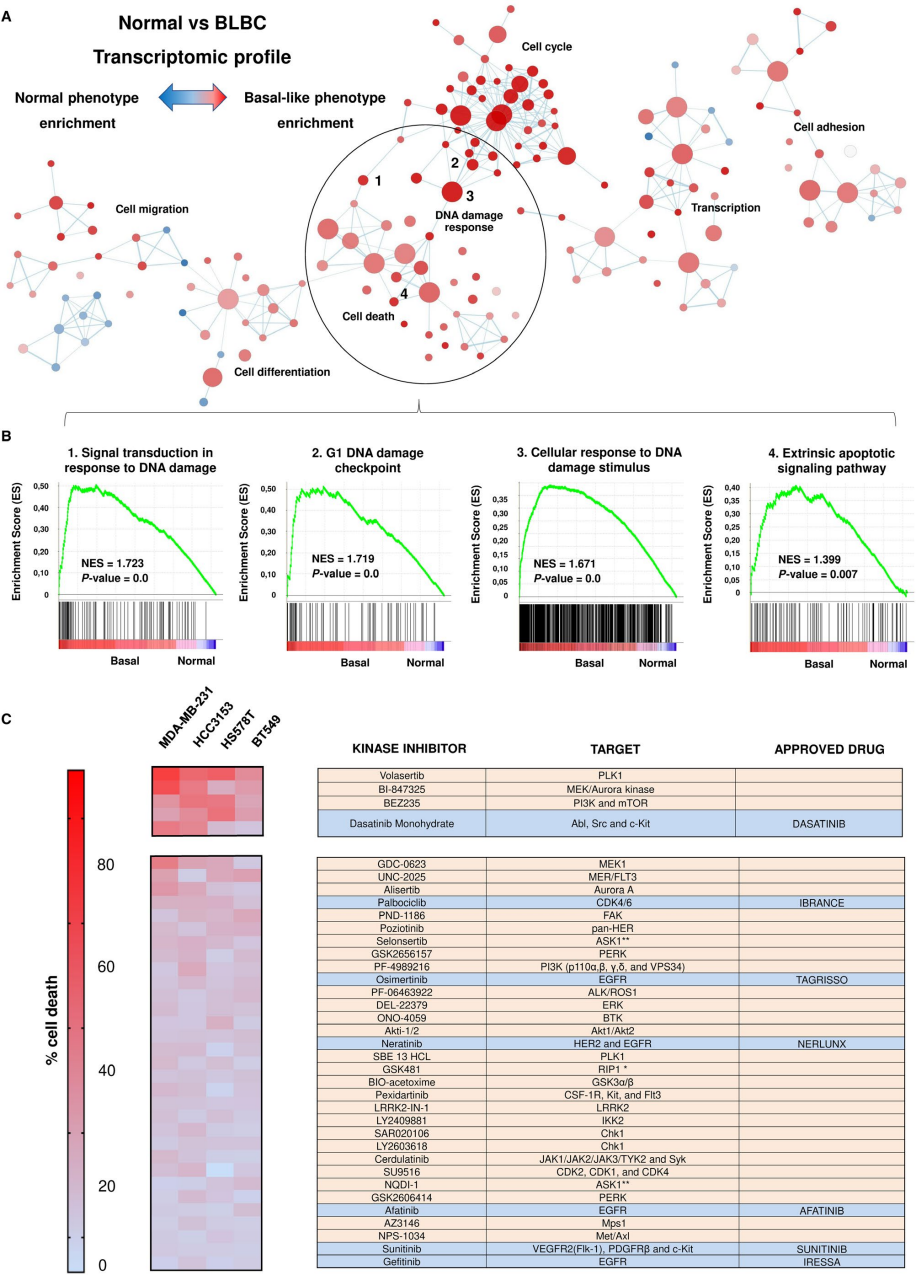
In silico analyses and pharmacological screening. A, Gene set enrichment expression network constructed by using Cytoscape after comparing normal and basal‐like tumours. A total of 178 different gene sets related with several cellular functions were represented. Blue‐red intensity of colour was determined by normalized enrichment score and defined the phenotype enrichment. B, Enrichment score profile and locations of gene set members on the rank ordered list of the four most altered DNA damage signatures. All of them were enriched for basal‐like phenotype. C, Antiproliferative effect of the kinase inhibitor library in TNBC cell lines. Cell death is represented in a heat map; red colour indicates more antiproliferative effect. MDA‐MB‐231, HS‐578T, BT549 and HCC3153 cells were treated with the indicated kinase inhibitor (100 nmol/L) for 3 d. Then, cell viability was determined by MTT assay. Molecular targets for each inhibitor and approved FDA drugs (blue), when existed, are listed in the annex table. Main biological functions (Uniprot) of FDA‐approved drugs are also indicated

To identify molecular alterations and druggable vulnerabilities in triple‐negative breast cancer, we took two different approaches. First, we performed gene expression analyses comparing normal breast with basal‐like tumours. To do so, we used several public available data sets (GSE21422, GSE26910, GSE3744, GSE65194 and GSE42568). Functional annotation analyses identified several altered pathways, including transcription, adhesion, cell cycle, differentiation, migration, DNA damage response and cell death (Figure 1A). We focused on these last two functions, as novel compounds against proteins within these alterations are currently in clinical development. Gene set enrichment analyses showed a marked up‐regulation of several of DNA damage‐response pathways, reinforcing the role of this alteration as pathogenic in this breast cancer subtype (Figure 1B). In addition to this genomic approach, we performed a pharmacologic screening on triple‐negative cell lines (MDA‐MB‐231, HS‐578T, BT549 and HCC3153) using agents against several cell signalling mediators involved in the previously described nodes (Figure 1C). We focused on dasatinib, a multikinase inhibitor, with effect against SRC, abl and c‐kit, among other kinases, that is approved in patients with chronic myeloid leukaemia (CML).^16^

### 3.2 The multikinase inhibitor dasatinib enhances the antitumoral effect of olaparib

Given the fact that DNA damage response was the most prominent function identified in the functional annotation analyses and that the PARP inhibitor olaparib is approved in breast cancer, we decided to explore the effect of the combination of dasatinib with this agent in TNBC cell lines (MDA‐MB‐231, HS‐578T, BT549 and HCC3153).

**Figure 2:**
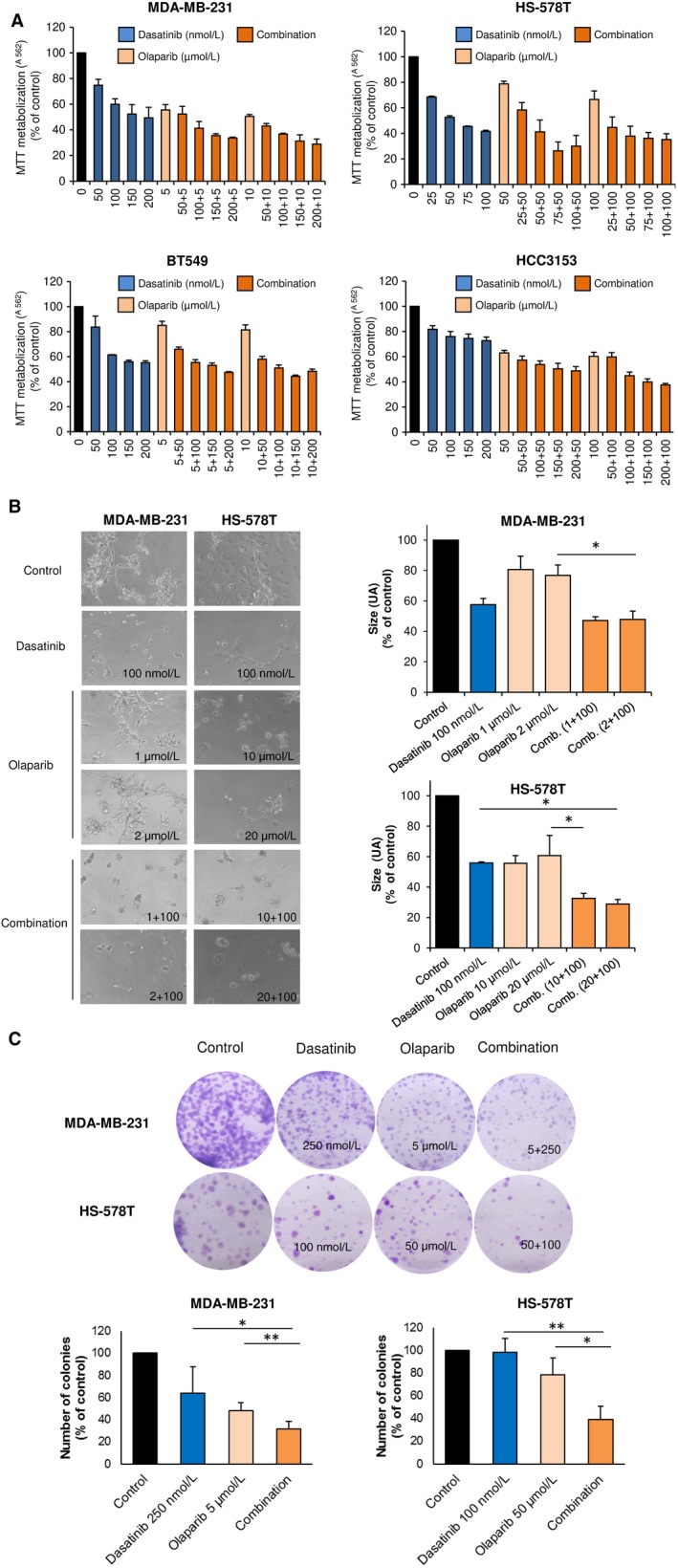
Antiproliferative and anti‐invasive effect of dasatinib‐olaparib combination in TNBC cells. A, Antiproliferative effect of dasatinib and olaparib combination in TNBC cells. MDA‐MB‐231, HS‐578T, BT549 and HCC3153 cells were incubated with increasing concentrations of dasatinib alone or in combination with olaparib indicated doses for 3 d and, then, cell viability was assessed by MTT assay. MTT metabolization is referred to control absorbance values. Results are shown as the mean of three independent experiment ± SD, each of them performed with triplicates. B, Invasion capacity of matrigel‐embedded invading cultures of MDA‐MB‐231 and HS‐578T is impaired with dasatinib and olaparib. Cells were grown in a semi‐solid matrigel matrix. Then, invading cultures were exposed to the indicated doses of the drugs, alone or in combination, for 3 d. Size is referred to area score as arbitrary length units (AU). Data of three independent experiments ± SD. C, Clonogenic formation of MDA‐MB‐231 and HS‐578T is reduced with the combination. Cells were treated (24 h) with the indicated doses. After, cells were reseeded (500 and 1000 cells, respectably). The colonies formed were fixed, stained and counted. Results were referred to control as percentage. Data of three independent experiments ± SD. *P < .05; **P < .01; ***P < .001

As can be seen in Figure 2A, increasing doses of dasatinib, in the nanoMolar range, in combination with a fix dose of olaparib, clearly augmented the effect of each agent given alone (Figure 2A). However, no synergistic interaction was found when we used a non‐tumorigenic epithelial cell line, such as HACAT cells. Of note, single treatment with dasatinib had an antiproliferative effect in this cell line, although to a less extent than in transformed cells. Little activity for olaparib as a single agent was observed (Figure S1).

Next, we confirmed this interaction performing studies on MDA‐MB‐231 and HS‐578T, the two most sensitive cell lines, using a semi‐solid media with Matrigel assay and clonogenic assays. As shown, combination impaired invading structures (Figure 2B) and colonies formation (Figure 2C). Besides, soft agar formation assays confirmed that single treatment with olaparib or the combination completely abolished anchorage‐independent growth of both cell lines (Figure S2). Of note, no mutations in the BRCA1 and BRCA2 genes are described in these cell lines except for BRCA1 in HCC3153 (Table S2), and no difference in response was observed (Figure 2A).

### 3.3 Pleiotropic effect of the combination on cell cycle and induction of apoptosis

**Figure 3:**
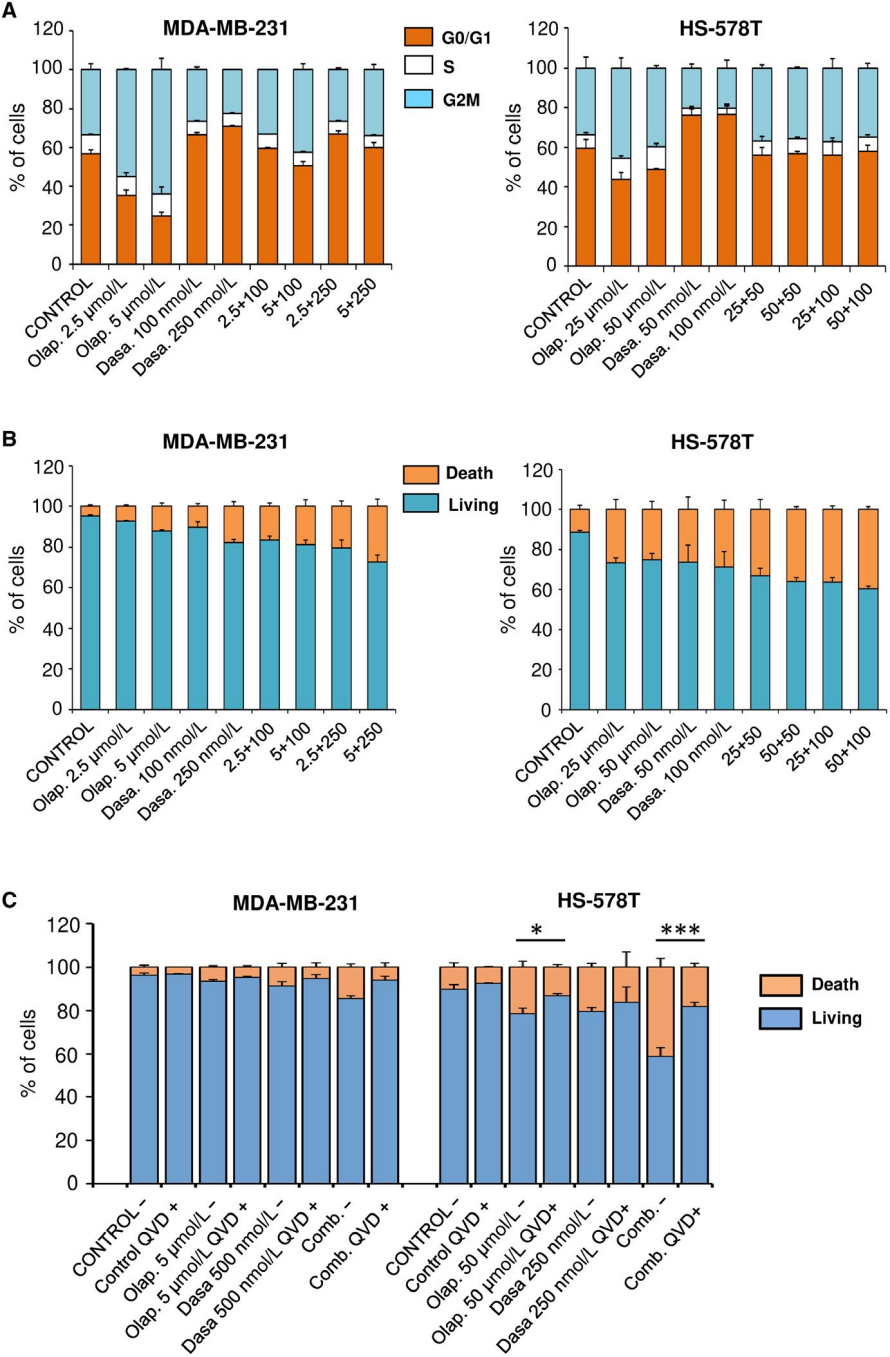
Cell death and cell cycle analysis by flow cytometry. A, Olaparib and dasatinib treatment leads to cell cycle arrest. MDA‐MB‐231 and HS‐578T were incubated the indicated doses of dasatinib, olaparib or both. After 24 h, cell cycle progression was examined by flow cytometry. Propidium iodide was used as DNA staining. Histogram shows the percentage of cells in the different phases of the cell cycle. B, Olaparib and dasatinib combination increases cell death. MDA‐MB‐231 and HS‐578T were incubated with the indicated doses of dasatinib, olaparib or both. After 72 h, apopototic activity was evaluated by flow cytometry. Annexin V was used as staining in combination with propidium iodide. Histograms present percentage of cells divided into living cells (AV‐, IP‐) vs apoptotic cells (AV+, PI‐ and AV+, PI+). C, Olaparib and dasatinib‐associated apoptosis is caspase‐dependent. MDA‐MB‐231 and HS‐578T were incubated with the indicated doses of dasatinib, olaparib or both, in presence or absence of pan‐caspase inhibitor QVD (10 μmol/L). After 72 h, apoptotic activity was evaluated by flow cytometry as explained in B*.* Results were show as living cells (AV‐, PI‐) vs apoptotic cells (AV+, PI‐ and AV+, PI+)

**Figure 4:**
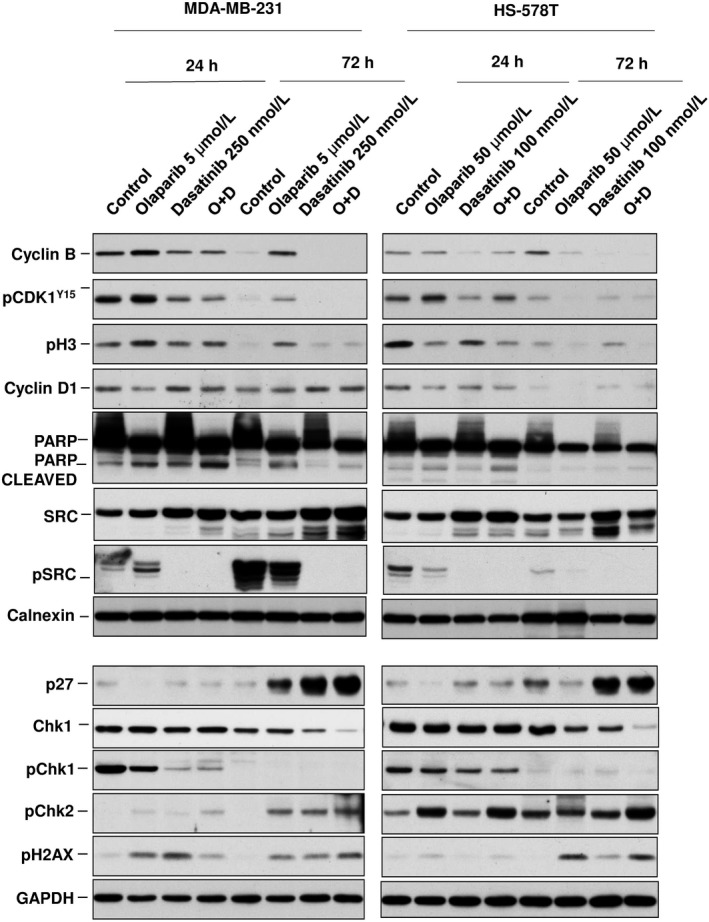
Biochemical analyses of proteins involved in cell cycle progression and cell death. Protein levels of Cyclin B, pCDK1(Y15), pH3, Cyclin D1, PARP and C‐PARP, p27, Chk1, pChk1, pChk2, pSrc, Src and pH2AX were evaluated in MDA‐MB‐231 and HS‐578T following dasatinib (250 and 100 nm, respectively) and olaparib (5 and 50 μmol/L) single and combination treatments (24 and 72 h) determined by Western blotting. Calnexin and GAPDH were used as loading control

To get insides into the mechanism of action, we evaluated the effect of both compounds, alone or combined, on cell cycle using MDA‐MB‐231 and HS‐578T cells. In both cell lines, olaparib induced G2/M cell cycle arrest while dasatinib increased G0/G1 checkpoint (Figure 3A). Of note, the combination produced a pleotropic effect with no clear action on any specific cell cycle phase, what is also a reflection of the asynchrony that exist among the population of cancer cells (Figure 3A). However, the combination produced a strong induction of apoptosis in both cell lines, probably due to the fact that each agent acted on a different cell cycle phase, altering cell cycle normal progression (Figure 3B). Next, we explored if the induction of apoptosis was dependent on caspases activation. To do so, we cultured the cell lines in the presence or absence of a pan‐caspase inhibitor (QVD) prior treatment with olaparib, dasatinib or the combination of both. As shown, a clear reduction in cell death was observed in the combination in the presence of the caspase inhibitor, demonstrating that the effect was mainly mediated by caspases (Figure 3C). In line with this, the combination of both agents induced PARP cleaved more clearly seen in MDA‐MB‐231 (Figure 4). This set of experiments reveals that each agent acted on different cell cycle phases inducing cell cycle arrest, and when added together, the combination was able to lead to a strong induction of apoptosis in a caspase‐dependent manner.

### 3.4 Effect of the combination on cell cycle mediators

We performed biochemical experiments to better characterize the mechanism of action of each agent alone or combined. Treatment with olaparib reduced the levels of Cyclin D and increased the phosphorylation of H3 in MD‐MB‐231, which is indicative of a reduction of cells in G1 and an arrest in mitosis, at 24 hours (Figure 4). This effect was not observed in HS‐578T. Exposure to dasatinib and the combination reduced the expression of pChk1 and increased pH2AX in both cell lines at 24 hours, what correlated with an induction of DNA damage. Of note, the phosphorylated form was not observed at 72 hours, probably due to a degradative effect of the drugs on Chk1. Conversely, pChk2 expression was increased. Total levels of Chk1 did not correlate with response (Figure S3). Dasatinib completely inhibit the activation of SRC alone and in combination with olaparib. An increase in p27 was produced by dasatinib and the combination at 72 hours (Figure 4). These findings suggest that the effect on cell cycle mediators is pleiotropic, affecting different components, as observed in the cell cycle analyses and varies depending on the cell line. Dasatinib was able to induce DNA damage therefore increasing the genetic instability.

### 3.5 Synergistic action in other tumour types

**Figure 5:**
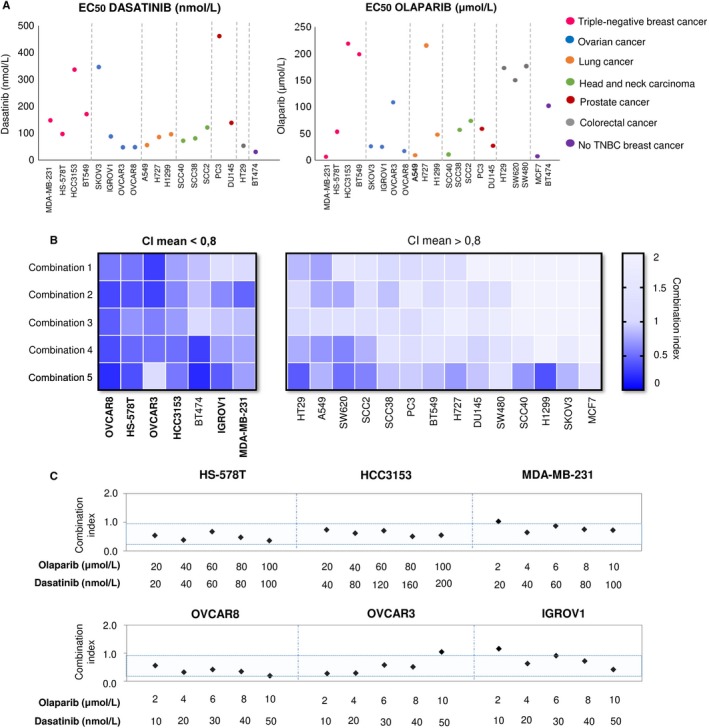
Action of dasatinib and olaparib alone or in combination in multiple solid tumours cell lines. A, Effect of dasatinib and olaparib in individual treatments. EC_50_ values of the different solid tumours cancer cell lines after single dasatinib or olaparib treatment (72 h). Ovarian cancer, lung cancer, head and neck squamous carcinoma, prostate cancer, colorectal cancer, TNBC and non‐TNBC cell lines were used and indicated with different colours. Dasatinib EC_50_ values of MCF7, SW620 and SW480 cell lines are highest than 200 nmol/L. Cell viability was determinate by MTT colorimetry assay. MTT metabolization was referred to control (%). EC_50_ values were obtained by GraphPad software. B, Effect of dasatinib and olaparib combination treatments. Combination index (CI) heat map of drug combinations in solid tumours cancer cell lines. CI for the different drug combinations were obtained using CalcuSyn program from viability values obtained in an MTT assay after 72 h of incubation with the drugs. Combination doses used are shown in Table S1 *.* CI values lower than 0.8 indicate synergistic action. C, Synergistic action of dasatinib and olaparib on TNBC cells and ovarian cancer cells. Synergistic antiproliferative effects of dasatinib and olaparib in TNBC cell lines (HS‐578T, HCC3153 and MDA‐MB‐231) and ovarian cell lines (OVCAR8, OVCAR3 and IGROV1). CI for the different drug combinations were obtained as described in B. Blue zone represents CI synergistic values for the different drug combinations

Finally, we aimed to explore the activity of both compounds alone or in combination in 18 cell lines representative of several tumour types, including ovarian, lung, prostate, colorectal cancer, head and neck carcinoma, and non–triple‐negative breast tumours. Dasatinib showed activity in most of the cell lines, including ovarian, lung, head and neck, colorectal and HER2 positive breast. Olaparib showed modest activity in all cell lines except in ovarian cancer (Figure 5A). Studies with both agents at different concentrations showed synergistic results in ovarian cancer cell lines (OVCAR8, OVCAR3 and IGROV1), triple negative (HS‐578T, HCC3153 and MDA‐MB‐231), and the HER2‐positive BT474 (Figure 5B). On the other hand, no synergistic interactions were observed for the rest of tumoural cells (Figure 5B and Table S3 for the description of the combinations used). The specific doses used in ovarian cancer and triple‐negative cell lines are listed in Figure 5C.

## 4 DISCUSSION

In the present article, we identify a synergistic interaction between the PARP inhibitor olaparib and the approved multikinase inhibitor dasatinib. This interaction was particularly active in triple‐negative breast and ovarian cancer cell lines. Olaparib has recently been approved for the treatment of these cancers with germline mutations of BRCA genes.^6^, ^7^, ^8^ Due to the limit number of therapeutic options for these tumours, the identification of novel combinations that can increase efficacy without overlapping toxicities is a main objective.

Gene expression analyses and functional annotation identified several deregulated functions, including DNA damage response as a druggable vulnerability. The use of gene set enrichment analyses to identify therapeutic options including treatment combinations has been explored before with successful results.^17^, ^18^ As mentioned, agents acting on DNA‐damaging response components have reached the clinical setting, like is the case of PARP inhibitors.^6^, ^7^, ^8^ In parallel a pharmacologic screening identified dasatinib as the most FDA‐approved active compound (80% inhibition of cell proliferation). Although other compounds of our library showed more activity, like the dual PI3K/mTOR inhibitor BEZ235, the MEK/Aurora kinase BI‐847325, or the PLK1 inhibitor volasertib, their antitumoural action has already been described in these indications and have not gained approval for use in humans, so their potential applicability to patients rested medical interest.^19^, ^20^, ^21^

In our experiments, exposure to increasing doses of dasatinib to a fix dose of olaparib augmented its efficacy in several triple‐negative cell lines, effect that was also observed using semi‐solid media with matrigel.

While the effect of dasatinib induced cell cycle arrest in G1 and olaparib in G2/M, the combination of both agents had a pleotropic effect on cell cycle. This can also be explained by the asynchrony division rate among cells. However, we clearly observed that the combination strongly augmented cell death. This finding suggests that acting on two different cell cycle phases can increase cell death; an effect also observed with other combinations.^22^ When exploring the mechanism involved in cell death, a caspase‐dependent action was identified.

The biochemical evaluation of the combination showed effects on different components that were slightly different between the two evaluated cell lines. However, differences in the mechanism of action of the same compound between cell lines have been described before and is a confirmation of the heterogeneity of tumour cells.^22^ Dasatinib‐inhibited pChk1 and increased pH2AX, markers of DNA damage, suggesting that this compound facilitates genetic instability sensitizing cancer cells to the action of PARP inhibitors. Finally, combinations of both agents induce cell death observing a clear increase in PARP cleaved. As observed, this mechanism could be at least partially depended on caspases. Of note, basal expression of Chk1 did not influence efficacy as did not the presence of mutations at BRCA1 gene in HCC3153. Interestingly, the observed effect was independent of the presence of deleterious mutations at BRCA genes. For instance, HCC3153 is a well‐characterized cell line with a deleterious mutation of BRCA1^23^ that leads to a truncated protein, but no differences in efficacy were observed.

A main limitation of the clinical development of PARP inhibitors is the overlapping of toxicities when combined with DNA damaging chemotherapies.^11^, ^12^, ^13^ In this context, combinations of olaparib with other compounds, like antiangiogenic agents, have also been evaluated and are in clinical development.^24^ Very recent combinations of PARP inhibitors include epigenetic agents like HDAC inhibitors or immune modulators like PD1 or PD‐L1 inhibitors.^25^, ^26^, ^27^, ^28^

Finally, we observed that the synergy observed with the combination of olaparib and dasatinib was not restricted to TNBC cell lines, but also extended to ovarian tumours, increasing the scenario of potential clinical indications. However, little effect was observed in other solid cancer cell models.

In conclusion, we describe a novel synergistic interaction between PARP inhibition and dasatinib in triple‐negative breast and ovarian cancer. As both drugs are approved for the treatment of human cancers, our results pave the way for the clinical development of these compounds in triple‐negative breast and ovarian tumours.

## CONFLICTS OF INTERESTS

The authors declare that they have no competing interests.

## AUTHORS' CONTRIBUTIONS

AO had the idea. AO and AP designed the experiments, supervised the execution and wrote the article. MN also wrote the article. VC, MN, CN, JM, EG, JP and MB performed the experiments. All authors approved the final version of the manuscript.

## Data Availability

Part of the data that support the findings of this study are publicly available in the Gene Expression Omnibus (GEO) repository, data set accession numbers: GSE21422, GSE26910, GSE3744, GSE65194, and GSE42568. All other data supporting the findings of this study are available within the article and its supplementary information files.
