# Supplementary figures and images for: Pharmacological screening and transcriptomic functional analyses identify a synergistic interaction between dasatinib and olaparib in triple‐negative breast cancer
Source: J Cell Mol Med. 2020 Feb 7;24(5):3117–27. doi: 10.1111/jcmm.14980 (PMC7077558; doi:10.1111/jcmm.14980)

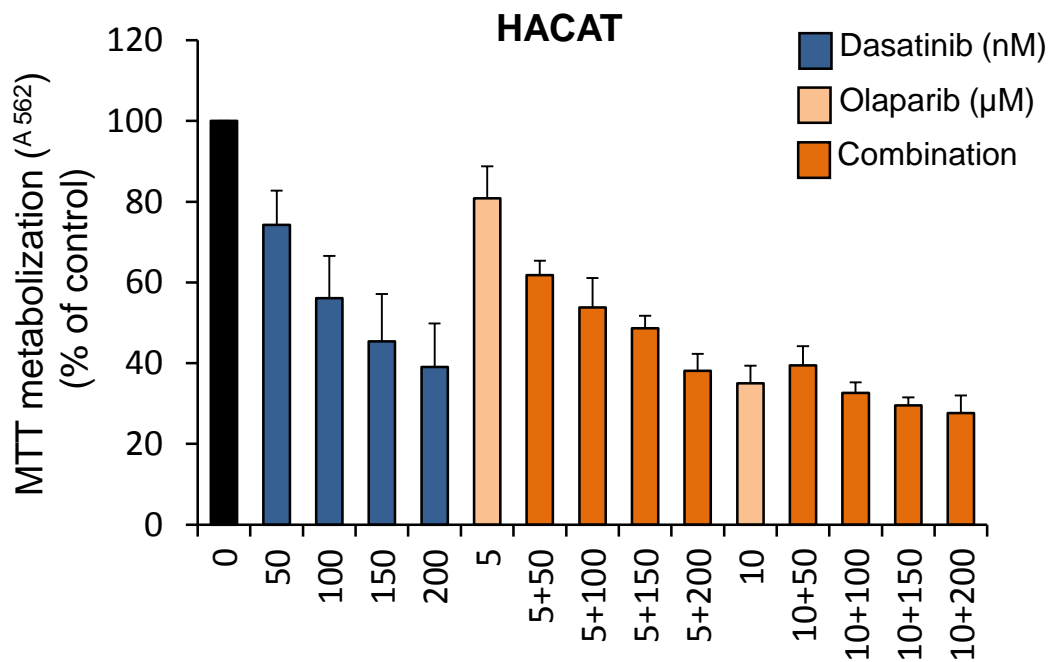

Supplementary Figure 1

Supplement: Supplementary file 1 [file JCMM-24-3117-s001.pdf]

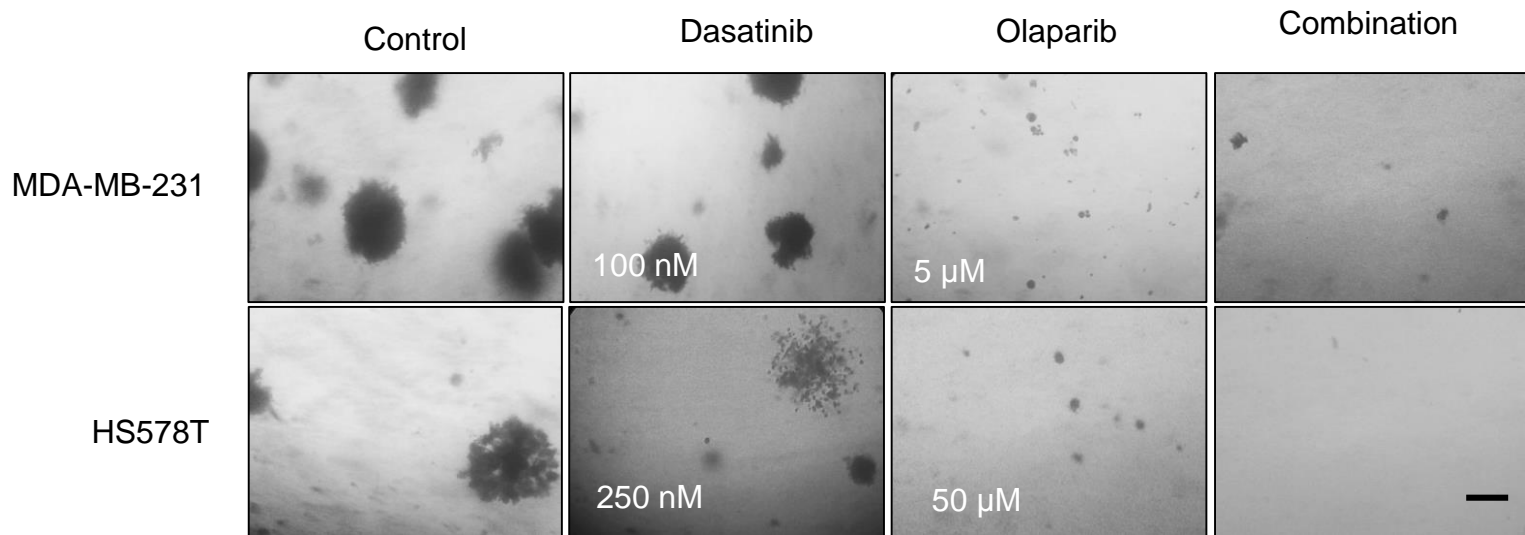

Supplementary Figure 2

Supplement: Supplementary file 2 [file JCMM-24-3117-s002.pdf]

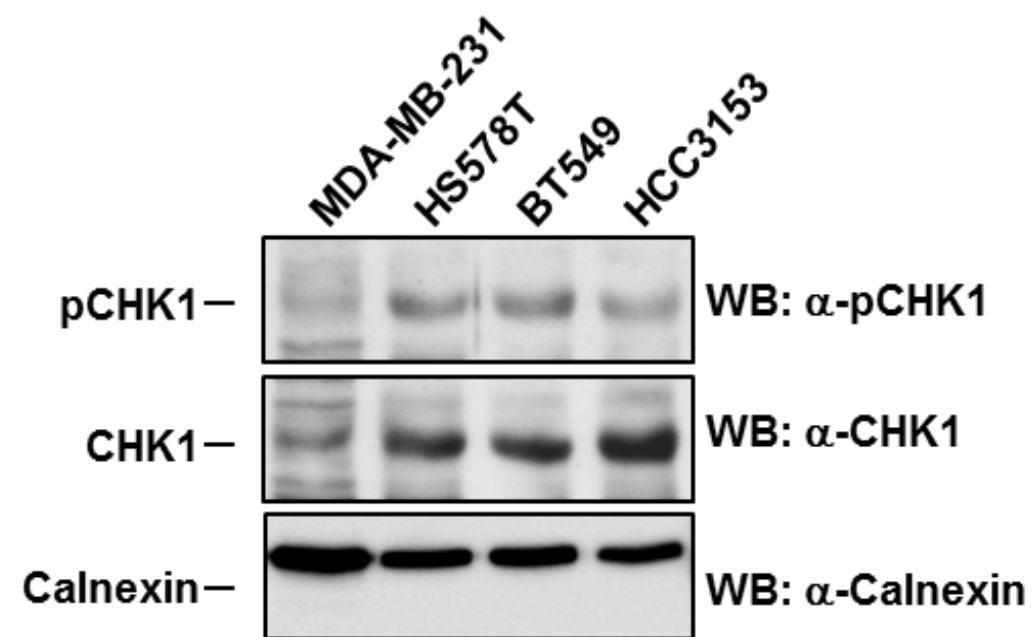

Supplementary figure 3

Supplement: Supplementary file 3 [file JCMM-24-3117-s003.pdf]
