# Supplementary material for: Pharmacological screening and transcriptomic functional analyses identify a synergistic interaction between dasatinib and olaparib in triple‐negative breast cancer
Source: J Cell Mol Med. 2020 Feb 7;24(5):3117–27. doi: 10.1111/jcmm.14980 (PMC7077558; doi:10.1111/jcmm.14980)
Supplement: Supplementary file 4 [file JCMM-24-3117-s004.pdf]

# Samples used for Breast Cancer Transcriptional Map

| 46 Normal Breast Samples | 73 Basal-like Samples | 29 Luminal A Samples | 30 Luminal B Samples | 39 HER2+ Samples |
|---|---|---|---|---|
| GSM1045191_N1_15_12_04.CEL | GSM1588970_BC16_ARN0076_s1h1s1_U133p2.CEL | GSM1589064_BC16_ARN0065_s1h1s1_U133p2.CEL | GSM1589093_BC16_ARN0129_s1h1s1_U133p2.CEL | GSM1588972_BC16_ARN0043_s1h1s1_U133p2.CEL |
| GSM1045192_N4_14_12_04.CEL | GSM1588971_BC04_ARN0025_s1h1s1_U133p2.CEL | GSM1589065_BC16_ARN0106_s1h1s1_U133p2.CEL | GSM1589094_BC16_ARN0046_s1h1s1_U133p2.CEL | GSM1588973_BC04_ARN0026_s1h1s1_U133p2.CEL |
| GSM1045193_N5_15_12_04.CEL | GSM1588974_BC04_ARN0027_s1h1s1_U133p2.CEL | GSM1589066_BC16_ARN0126_s1h1s1_U133p2.CEL | GSM1589095_BC16_ARN0115_s1h1s1_U133p2.CEL | GSM1588981_BC04_ARN0003_s1h1s1_U133p2.CEL |
| GSM1045194_N6_14_12_04.CEL | GSM1588975_BC04_ARN0028_s1h1s1_U133p2.CEL | GSM1589067_BC16_ARN0080_s1h1s1_U133p2.CEL | GSM1589097_BC16_ARN0054_s1h1s1_U133p2.CEL | GSM1588989_BC16_ARN0103_s1h1s1_U133p2.CEL |
| GSM1045195_P1_15_12_04.CEL | GSM1588976_BC04_ARN0001_s1h1s1_U133p2.CEL | GSM1589068_BC16_ARN0036_s1h1s1_U133p2.CEL | GSM1589098_BC16_ARN0074_s1h1s1_U133p2.CEL | GSM1588990_BC04_ARN0005_s1h1s1_U133p2.CEL |
| GSM1045196_P10_26_1_05.CEL | GSM1588977_BC16_ARN0062_s1h1s1_U133p2.CEL | GSM1589069_BC16_ARN0105_s1h1s1_U133p2.CEL | GSM1589099_BC16_ARN0066_s1h1s1_U133p2.CEL | GSM1588991_BC16_ARN0063_s1h1s1_U133p2.CEL |
| GSM1045197_P11_25_1_05.CEL | GSM1588978_BC04_ARN0002_s1h1s1_U133p2.CEL | GSM1589070_BC16_ARN0092_s1h1s1_U133p2.CEL | GSM1589101_BC16_ARN0123_s1h1s1_U133p2.CEL | GSM1588992_BC04_ARN0036_s1h1s1_U133p2.CEL |
| GSM1045198_P12_25_1_05.CEL | GSM1588979_BC04_ARN0029_s1h1s1_U133p2.CEL | GSM1589071_BC16_ARN0125_s1h1s1_U133p2.CEL | GSM1589103_BC16_ARN0087_s1h1s1_U133p2.CEL | GSM1588997_BC04_ARN0040_s1h1s1_U133p2.CEL |
| GSM1045199_P13_25_1_05.CEL | GSM1588980_BC04_ARN0030_s1h1s1_U133p2.CEL | GSM1589072_BC16_ARN0112_s1h1s1_U133p2.CEL | GSM1589105_BC16_ARN0041_s1h1s1_U133p2.CEL | GSM1588998_BC16_ARN0050_s1h1s1_U133p2.CEL |
| GSM1045200_P14_25_1_05.CEL | GSM1588982_BC16_ARN0035_s1h1s1_U133p2.CEL | GSM1589073_BC16_ARN0037_s1h1s1_U133p2.CEL | GSM1589107_BC16_ARN0067_s1h1s1_U133p2.CEL | GSM1588999_BC04_ARN0041_s1h1s1_U133p2.CEL |
| GSM1045201_P18_25_1_05.CEL | GSM1588983_BC04_ARN0031_s1h1s1_U133p2.CEL | GSM1589074_BC16_ARN0079_s1h1s1_U133p2.CEL | GSM1589109_BC16_ARN0075_s1h1s1_U133p2.CEL | GSM1589000_BC16_ARN0097_s1h1s1_U133p2.CEL |
| GSM1045202_P21_25_1_05.CEL | GSM1588984_BC04_ARN0032_s2h1s1_U133p2.CEL | GSM1589075_BC16_ARN0132_s1h1s1_U133p2.CEL | GSM1589110_BC16_ARN0136_s1h1s1_U133p2.CEL | GSM1589001_BC04_ARN0006_s1h1s1_U133p2.CEL |
| GSM1045203_P3_22_12_04.CEL | GSM1588985_BC04_ARN0033_s2h2s1_U133p2.CEL | GSM1589076_BC16_ARN0133_s1h1s1_U133p2.CEL | GSM1589111_BC16_ARN0040_s1h1s1_U133p2.CEL | GSM1589002_BC04_ARN0042_s1h1s1_U133p2.CEL |
| GSM1045204_P4_14_12_04.CEL | GSM1588986_BC16_ARN0110_s1h1s1_U133p2.CEL | GSM1589077_BC16_ARN0058_s1h1s1_U133p2.CEL | GSM1589112_BC16_ARN0089_s1h1s1_U133p2.CEL | GSM1589006_BC16_ARN0131_s1h1s1_U133p2.CEL |
| GSM1045205_P5_21_12_04.CEL | GSM1588987_BC04_ARN0004_s1h1s1_U133p2.CEL | GSM1589078_BC16_ARN0119_s1h1s1_U133p2.CEL | GSM1589113_BC16_ARN0088_s1h1s1_U133p2.CEL | GSM1589007_BC04_ARN0045_s1h1s1_U133p2.CEL |
| GSM1045206_P8_25_1_05.CEL | GSM1588988_BC04_ARN0034_s2h1s1_U133p2.CEL | GSM1589079_BC16_ARN0086_s1h1s1_U133p2.CEL | GSM1589114_BC16_ARN0093_s1h1s1_U133p2.CEL | GSM1589008_BC04_ARN0046_s1h1s1_U133p2.CEL |
| GSM1045207_P9_25_1_05.CEL | GSM1588993_BC16_ARN0102_s1h1s1_U133p2.CEL | GSM1589080_BC16_ARN0064_s1h1s1_U133p2.CEL | GSM1589115_BC16_ARN0059_s1h1s1_U133p2.CEL | GSM1589013_BC04_ARN0048_s2h2s1_U133p2.CEL |
| GSM1589130_BC16_ARN0018_s1h1s1_U133p2.CEL | GSM1588994_BC04_ARN0038_s1h1s1_U133p2.CEL | GSM1589081_BC16_ARN0071_s1h1s1_U133p2.CEL | GSM1589116_BC16_ARN0094_s1h1s1_U133p2.CEL | GSM1589018_BC16_ARN0118_s1h2s1_U133p2.CEL |
| GSM1589132_BC16_ARN0028_s1h1s1_U133p2.CEL | GSM1588995_BC16_ARN0096_s1h1s1_U133p2.CEL | GSM1589082_BC16_ARN0072_s1h1s1_U133p2.CEL | GSM1589117_BC16_ARN0135_s1h1s1_U133p2.CEL | GSM1589019_BC04_ARN0013_s1h1s1_U133p2.CEL |
| GSM1589135_BC16_ARN0024_s1h1s1_U133p2.CEL | GSM1588996_BC04_ARN0039_s1h1s1_U133p2.CEL | GSM1589083_BC16_ARN0051_s1h1s1_U133p2.CEL | GSM1589118_BC16_ARN0083_s1h1s1_U133p2.CEL | GSM1589020_BC04_ARN0051_s4h1s1_U133p2.CEL |
| GSM1589136_BC16_ARN0025_s1h1s1_U133p2.CEL | GSM1589003_BC04_ARN0043_s1h1s1_U133p2.CEL | GSM1589084_BC16_ARN0111_s1h1s1_U133p2.CEL | GSM1589119_BC16_ARN0122_s1h1s1_U133p2.CEL | GSM1589021_BC04_ARN0052_s1h1s1_U133p2.CEL |
| GSM1589139_BC16_ARN0033_s1h1s1_U133p2.CEL | GSM1589004_BC04_ARN0007_s1h1s1_U133p2.CEL | GSM1589085_BC16_ARN0099_s1h1s1_U133p2.CEL | GSM1589120_BC16_ARN0053_s1h1s1_U133p2.CEL | GSM1589022_BC16_ARN0077_s1h1s1_U133p2.CEL |
| GSM1589142_BC16_ARN0027_s1h1s1_U133p2.CEL | GSM1589005_BC04_ARN0044_s2h1s1_U133p2.CEL | GSM1589086_BC16_ARN0057_s1h1s1_U133p2.CEL | GSM1589121_BC16_ARN0068_s1h1s1_U133p2.CEL | GSM1589023_BC04_ARN0053_s1h1s1_U133p2.CEL |
| GSM1589144_BC16_ARN0026_s1h1s1_U133p2.CEL | GSM1589009_BC16_ARN0049_s1h1s1_U133p2.CEL | GSM1589087_BC16_ARN0104_s1h1s1_U133p2.CEL | GSM1589122_BC16_ARN0048_s1h1s1_U133p2.CEL | GSM1589025_BC04_ARN0056_s1h1s1_U133p2.CEL |
| GSM1589145_BC16_ARN0030_s1h1s1_U133p2.CEL | GSM1589010_BC04_ARN0008_s1h1s1_U133p2.CEL | GSM1589088_BC16_ARN0113_s1h1s1_U133p2.CEL | GSM1589123_BC16_ARN0101_s1h1s1_U133p2.CEL | GSM1589026_BC04_ARN0014_s1h1s1_U133p2.CEL |
| GSM1589148_BC16_ARN0019_s1h1s1_U133p2.CEL | GSM1589011_BC04_ARN0047_s2h1s1_U133p2.CEL | GSM1589089_BC16_ARN0045_s1h1s1_U133p2.CEL | GSM1589124_BC16_ARN0116_s1h1s1_U133p2.CEL | GSM1589027_BC04_ARN0015_s1h1s1_U133p2.CEL |
| GSM1589150_BC16_ARN0029_s1h1s1_U133p2.CEL | GSM1589012_BC04_ARN0009_s1h1s1_U133p2.CEL | GSM1589090_BC16_ARN0098_s1h1s1_U133p2.CEL | GSM1589125_BC16_ARN0082_s1h1s1_U133p2.CEL | GSM1589030_BC04_ARN0016_s1h1s1_U133p2.CEL |
| GSM1589151_BC16_ARN0020_s1h1s1_U133p2.CEL | GSM1589014_BC16_ARN0084_s1h1s1_U133p2.CEL | GSM1589091_BC16_ARN0078_s1h1s1_U133p2.CEL | GSM1589126_BC16_ARN0060_s1h1s1_U133p2.CEL | GSM1589033_BC04_ARN0059_s1h1s1_U133p2.CEL |
| GSM535613_wke-cs-h11-ges22906re-040608-u133p-2.CEL | GSM1589015_BC04_ARN0010_s1h1s1_U133p2.CEL | GSM1589092_BC16_ARN0085_s1h1s1_U133p2.CEL | GSM1589127_BC16_ARN0137_s1h1s1_U133p2.CEL | GSM1589034_BC04_ARN0061_s2h1s1_U133p2.CEL |
| GSM535614_wke-cs-h12-ges22806li-040608-u133p-2.CEL | GSM1589016_BC04_ARN0049_s1h1s1_U133p2.CEL | | GSM1589128_BC16_ARN0100_s1h1s1_U133p2.CEL | GSM1589044_BC04_ARN0018_s1h1s1_U133p2.CEL |
| GSM535615_wke-cs-h13-ges19906-040608-u133p-2.CEL | GSM1589017_BC04_ARN0050_s1h1s1_U133p2.CEL | | | GSM1589046_BC04_ARN0021_s1h1s1_U133p2.CEL |
| GSM535616_wke-cs-h14-ges171006-040608-u133p-2.CEL | GSM1589024_BC04_ARN0054_s1h1s1_U133p2.CEL | | | GSM1589047_BC16_ARN0070_s1h1s1_U133p2.CEL |
| GSM535617_wke-cs-h15-ges301006-040608-u133p-2.CEL | GSM1589028_BC16_ARN0124_s1h1s1_U133p2.CEL | | | GSM1589048_BC04_ARN0022_s1h1s1_U133p2.CEL |
| GSM662768.CEL | GSM1589029_BC04_ARN0057_s1h1s1_U133p2.CEL | | | GSM1589049_BC04_ARN0023_s1h1s1_U133p2.CEL |
| GSM662770.CEL | GSM1589031_BC16_ARN0042_s1h1s1_U133p2.CEL | | | GSM1589050_BC04_ARN0071_s2h1s1_U133p2.CEL |
| GSM662772.CEL | GSM1589032_BC04_ARN0017_s1h1s1_U133p2.CEL | | | GSM1589051_BC04_ARN0072_s1h1s1_U133p2.CEL |
| GSM662774.CEL | GSM1589035_BC16_ARN0055_s1h1s1_U133p2.CEL | | | GSM1589052_BC04_ARN0073_s1h1s1_U133p2.CEL |
| GSM662776.CEL | GSM1589036_BC04_ARN0063_s1h1s1_U133p2.CEL | | | GSM1589053_BC04_ARN0074_s1h1s1_U133p2.CEL |
| GSM662778.CEL | GSM1589037_BC04_ARN0064_s1h1s1_U133p2.CEL | | | GSM1589057_BC04_ARN0035_s1h1s1_U133p2.CEL |
| GSM85513.CEL | GSM1589038_BC04_ARN0065_s1h1s1_U133p2.CEL | | | |
| GSM85514.CEL | GSM1589039_BC16_ARN0069_s1h1s1_U133p2.CEL | | | |
| GSM85515.CEL | GSM1589040_BC04_ARN0020_s1h1s1_U133p2.CEL | | | |
| GSM85516.CEL | GSM1589041_BC04_ARN0066_s1h1s1_U133p2.CEL | | | |
| GSM85517.CEL | GSM1589042_BC04_ARN0067_s1h1s1_U133p2.CEL | | | |
| GSM85518.CEL | GSM1589043_BC04_ARN0069_s1h1s1_U133p2.CEL | | | |
| GSM85519.CEL | GSM1589045_BC04_ARN0011_s1h1s1_U133p2.CEL | | | |
| | GSM1589054_BC16_ARN0130_s1h1s1_U133p2.CEL | | | |
| | GSM1589055_BC04_ARN0070_s1h1s1_U133p2.CEL | | | |
| | GSM1589056_BC04_ARN0068_s1h1s1_U133p2.CEL | | | |
| | GSM1589058_BC16_ARN0117_s1h1s1_U133p2.CEL | | | |
| | GSM1589059_BC04_ARN0037_s1h1s1_U133p2.CEL | | | |
| | GSM1589060_BC04_ARN0055_s1h1s1_U133p2.CEL | | | |
| | GSM1589061_BC04_ARN0058_s1h1s1_U133p2.CEL | | | |
| | GSM1589062_BC04_ARN0019_s1h1s1_U133p2.CEL | | | |
| | GSM1589063_BC04_ARN0062_s1h1s1_U133p2.CEL | | | |
| | GSM85473.CEL | | | |
| | GSM85474.CEL | | | |
| | GSM85475.CEL | | | |
| | GSM85476.CEL | | | |
| | GSM85477.CEL | | | |
| | GSM85478.CEL | | | |
| | GSM85479.CEL | | | |
| | GSM85480.CEL | | | |
| | GSM85481.CEL | | | |
| | GSM85482.CEL | | | |
| | GSM85483.CEL | | | |
| | GSM85484.CEL | | | |
| | GSM85485.CEL | | | |
| | GSM85486.CEL | | | |
| | GSM85487.CEL | | | |
| | GSM85488.CEL | | | |
| | GSM85489.CEL | | | |
| | GSM85490.CEL | | | |

Supplementary Table 1
