# Supplementary material for: Pharmacological screening and transcriptomic functional analyses identify a synergistic interaction between dasatinib and olaparib in triple‐negative breast cancer
Source: J Cell Mol Med. 2020 Feb 7;24(5):3117–27. doi: 10.1111/jcmm.14980 (PMC7077558; doi:10.1111/jcmm.14980)
Supplement: Supplementary file 5 [file JCMM-24-3117-s005.pdf]

Cell line	Type of cancer	BRCA1 status	BRCA2 status
MDA-MB-231	Triple negative breast cancer	Wild type	Wild type
HS-578T		Wild type	Wild type
HCC3153		In Frame insertion	Wild type
BT549		Wild type	Wild type
SKOV3	Ovarian cancer	Wild type	Wild type
IGROV1		Wild type	Silent/ Missense mutation
OVCAR3		Wild type	Wild type
OVCAR8		Wild type	Wild type
A549	Lung cancer	Wild type	Wild type
H727		Missense mutation	Wild type
H1299		Wild type	Wild type
SCC40	Head and neck carcinoma	Wild type	Wild type
SCC38		Wild type	Wild type
SCC2		Wild type	Wild type
PC3	Prostate cancer	Wild type	Wild type
DU145		Missense mutation	Missense mutation
HT29	Colorectal cancer	Wild type	Wild type
SW620		Wild type	Wild type
SW480		Wild type	Wild type
MCF7	No TNBC breast cancer	Wild type	Wild type
BT474		Wild type	Nonsense mutation

Supplmentary table 2
