# Supplementary material for: Pharmacological screening and transcriptomic functional analyses identify a synergistic interaction between dasatinib and olaparib in triple‐negative breast cancer
Source: J Cell Mol Med. 2020 Feb 7;24(5):3117–27. doi: 10.1111/jcmm.14980 (PMC7077558; doi:10.1111/jcmm.14980)
Supplement: Supplementary file 6 [file JCMM-24-3117-s006.pdf]

Combination: Olaparib (µM) + Dasatinib (nM)

Cell line	COMB.1	COMB.2	COMB.3	COMB.4	COMB.5
A549, SKOV3, OVCAR3, OVCAR8, IGROV1 y SCC40	2+10	4+20	6+30	8+40	10+50
MDA-MB-231 y DU145	2+20	4+40	6+60	8+80	10+100
MCF7	2+40	4+80	6+120	8+160	10+200
H1299 y SCC38	4+20	8+40	12+60	16+80	20+100
HT29	10+10	20+20	30+30	40+40	50+50
SCC2	10+20	20+40	30+60	40+80	50+100
PC3	10+40	20+80	30+120	40+160	50+200
BT474	20+10	40+20	60+30	80+40	100+50
HS-578T, BT549 y H727	20+20	40+40	60+60	80+80	100+100
HCC3135, SW480 y SW620	20+40	40+80	60+120	80+160	100+200

Supplementary table 3
